# Mastitis in goat: A review of etiology, epidemiology, economic impact, and public health concerns

**DOI:** 10.1016/j.onehlt.2025.101131

**Published:** 2025-07-08

**Authors:** Abebe Tibebu, Yechale Teshome, Habtamu Tamrat, Adane Bahiru

**Affiliations:** a Sekota Dryland Agricultural Research Center, Sekota, Ethiopia; b Bahirdar University, School of Animal Science and Veterinary Medicine, Bahir Dar, Ethiopia

**Keywords:** Antimicrobial resistance, Causes, Goats, Mastitis, Public health

## Abstract

Mastitis is a multifactorial disease of mammals that has substantial implications on production, welfare, and public health, but it is far less studied in goats. This study reviewed the causes, diagnostic tests, economic and public health impacts, and prevalence of mastitis in goats. The review revealed that mastitis in goats could be clinical (CM) or subclinical (SCM). Bacteria, such as *Staphylococcus*, *Streptococcus*, *E. coli*, and *Mycoplasma*, are the main cause of mastitis in goats. Mastitis in goats is influenced by intrinsic factors like breed, parity, age, and lactation stage, and extrinsic factors like pathogens, environment, and management. Somatic cell count and the California mastitis test are common diagnostic tests for SCM, but their reliability is questionable due to the broader range of somatic cells in goat milk. Dry period therapy and early interventions are the best options for mastitis treatment and control methods, while temporal milking stops can enhance immunity. Mastitis can affect animal welfare, milk yield, quality, marketability, and public health due to disease transmission and intoxication. The pooled prevalence of mastitis was found to be 36 % (95 % CI: 25–50). The prevalence of CM was often less than 5 % and occurred sporadically. In contrast, SCM was the most prevalent form (prevalence ranges from 30 % to 50 %). In conclusion, despite goats being the foundation of financial stability and food security in rural households, and with an estimated prevalence comparable to cows, mastitis research is frequently overlooked. Given goats' importance in rural households' economies, there is a need to shift research priorities and strengthen multidisciplinary collaborations to address zoonotic diseases associated with mastitis, at the intersection of animal, environmental, and human health.

## 1 Introduction

The domestic goat (*Capra hircus*) is one of the oldest domesticated farm animals. More than 90 % of the goat population resides in Asia and Africa, with India, China, Pakistan, Nigeria, Bangladesh, and Ethiopia holding the majority share. France, Italy, Greece, and Spain dominate Europe's goat population and are leaders in global milk production per goat [1].

Goat farming provides sociocultural, economic, and disaster resilience benefits for rural and low-income farmers. It generates income through sales, savings, or asset accumulation; serves as insurance against economic uncertainties; and functions as a low-risk economic stabilizer [2]. Goat farming requires minimal initial investment, management, and maintenance, making it easily accessible to individuals or households with limited financial resources. The rising global demand for dairy products leads to a growing preference for dairy goat farming among landless and female-headed households [3,4]. Moreover, the natural ability of goats to withstand extreme conditions and adapt to challenging environments makes them preferred over cattle in arid and harsh climates [5].

Goats are the third largest contributor to global milk production, after cows and buffaloes. Goat milk accounted for 55 % of total milk production in Bangladesh, 52 % in Somalia, 43 % in Mali, 29 % in Indonesia, and 26 % in Greece. Besides, milk products like cheese, yogurt, and milk powder have seen a growing demand worldwide [1]. Furthermore, goats efficiently convert low-nutrition feed into high-quality milk enriched with calcium, potassium, protein, and vitamins. Singh [6] highlighted that goat milk has health benefits for treating cardiac diseases, mouth ulcers, and dengue fever. Additionally, goat milk has lower levels of casein, a substance that can trigger lactoglobulin allergies, compared to cow.

Although the goat population has grown steadily over the last three decades, milk production has not improved. The cause of this stagnation is unidentified, but contributing factors include a high prevalence of diseases that cause production losses, high mortality rates, poor growth, low fertility, and weight loss [4].

Mastitis, an inflammation of the mammary glands primarily caused by pathogens, is a substantial challenge in worldwide dairy production [7]. This inflammatory disease damages the mammary gland's milk-secreting cells, causing changes in its function and structure, as well as disruptions in milk production [8,9,10,11]. Dairy farmers are over-concerned about mastitis due to the huge financial losses it incurs, reduced farm returns, and costs of treating affected animals [12].

## 2 Types of mastitis

Mastitis is broadly classified into two: clinical mastitis (CM) and subclinical mastitis (SCM), based on visible clinical signs and presentations. Additionally, mastitis can be further categorized as contagious, which spreads from one animal to another, or environmental, originating from the surroundings. The most severe form of clinical mastitis is gangrenous mastitis, the common cause of unwanted culling in intensive farming [13,14].

### 2.1 Clinical mastitis

Clinical mastitis is characterized by visible mammary gland inflammatory signs such as swelling, localized heat and discoloration, pain upon palpation, and systemic signs like fever [15]. Milk from mastitic goats shows abnormalities like clots, discoloration, and reduced milk yield [16,11]. Clinical mastitis can cause lameness and altered gait due to udder discomfort. It can also progress to gangrene or septicemia and can be fatal [17]. Certain contagious bacteria, including *Mycoplasma mycoides*, and *Staphylococcus* spp., are responsible for sudden CM outbreaks in intensive production systems [17].

Clinical mastitis in goats occurs sporadically, with an annual incidence rate of less than 5 %. Studies have consistently shown a similar trend in different countries, including Algeria (3.55 %; [18]), Brazil (2.78 %; [19]), Indonesia (1.82 %; [20]).

### 2.2 Subclinical mastitis

Subclinical mastitis is defined by the absence of obvious clinical signs/udder abnormalities and can only be detected using diagnostic procedures. Subclinical mastitis accounts for 30–50 % of mastitis cases in goats, but it is often misestimated due to unclear cutoff points for common diagnostic tests in goats. Milk somatic cell counts greater than 1 × 10^6^ cells/ml is often considered an indication of intramammary inflammation. However, SCM affects later stages of lactation, as it spreads invisibly within the herd and poses a risk of latent infections [15,21,22].

## 3 Pathogenesis of mastitis

Mastitis is an inflammatory response of the mammary gland triggered by either noninfectious causes or infectious causes. The precise pathogenesis, when caused by different pathogens, is not clearly understood. It is determined by interactions among the environment, host, and causative agents [23,20].

Pathogens must enter the teat canal and overcome the physical barriers of keratin, glandular lining cells, and evading milk flow in order to cause IMI. The udder massaging and stripping create a vacuum, weakening the teat orifice's protective keratin. This allows bacteria to passively diffuse and attach to gland lining cells. Adhesion is considered a prerequisite and critical early step for intramammary infection development, followed by invasion and multiplication [23].

In response to pathogen invasion and multiplication, the “blood-milk barrier,” the lining structure between the blood vessel and the alveoli of milk-secreting cells, increases permeability, allowing humoral cells (neutrophils and lymphocytes with cytokines and chemokines) and antibodies to leak into the alveolar lumen and then into the milk. This progress is then marked by pain as immune response cells work, and heat, redness, and swelling are caused by fluid leaking from dilated blood vessels with increased permeability [24].

The immunity response may fail to control infection progression effectively when a high bacterial load enters the udder or when bacteria develop resistance mechanisms, such as altering receptor cells, acquiring resistance genes, or forming biofilms and degenerative substances. Biofilms are three-dimensional structures containing bacterial cells enveloped in a protective polymeric matrix, which renders them highly resistant to antimicrobials and the human immune system [25,26]. Consequently, physiological, anatomical, histopathological, and chemical changes occur in the udder and milk. The presence of humoral cells and fragmented epithelial cells, mitochondrial DNA, and cytoplasmic globules in milk secretion increases SCC, which is an indicator of IMI. Pathogens may also spread through the circulation, such as mycoplasmosis and lentiviral infections.

## 4 Causes of mastitis

Mastitis pathogens originate from two sources: infected animals and the environment. Contagious mastitis pathogens spread during milking via hands, towels, or equipment, while environmental mastitis pathogens splash from contaminated soil, manure, or bedding and are therefore linked to unsanitary conditions [27]. Table 1 summarizes the overview of mastitis causes and risk factors.

**Table 1:** Overview of mastitis causes and risk factors in goats.

Category	Cause and risk factors
Infectious Causes (Contagious)	*Staphylococcus aureus*, *Streptococcus agalactiae*, *Mycoplasma mycoides*, *Corynebacterium*, *Mycobacterium*, CAEV, *Maedi Visna Virus, Papilloma Virus*
Infectious Causes (Environmental)	*E. coli*, *Klebsiella*, *Streptococcus uberis*, *Streptococcus dysgalactiae*, Non-aureus *Staphylococcus*, *Micrococcus*, *Enterococcus*, *Pseudomonas*, *Serratia*, Fungal
Non-Infectious Causes	Physical trauma, metabolic disorder, bruising, cuts, burns, dryness, over-accumulation of milk in udder, extreme hot/cold temperature
Host Risk Factors	Age, parity, lactation stage, breed/genetics, estrus and pregnancy, twinning, hormonal factors, browsing nature of goat on thorns
Environmental Risk Factors	Unhygienic milking, lack of teat disinfection, muddy/wet bedding, abrasive fences/floors, excessive teat massage, under-milking, faulty milking machine pressure

### 4.1 Noninfectious causes of mastitis

Noninfectious causes of mastitis include genetics, environmental irritants, trauma, poor milking hygiene, and hormone imbalances. Extrinsic factors like tick infestations, mange mites, or teat injuries are common triggers of noninfectious mastitis [28].

### 4.2 Infectious mastitis

Mastitis can be classified as either environmental or contagious depending on its origin. Contagious mastitis is mainly spread through contact with contaminated equipment or infected udders. Environmental mastitis is often associated with wet or muddy conditions, dirty bedding, and insufficient barn cleanliness [29].

### 4.3 Bacterial causes

Mastitis is caused by a variety of pathogens, with bacteria being the most common in goats. *Staphylococcus* spp., a gram-positive, grape-like cocci commensal to udder and skin, is the leading cause of mastitis. *Staphylococcus* spp. were previously classified into two types based on their ability to react with coagulase: coagulase-positive (CP) and coagulase-negative (CN). A recent species classification was made as *Staphylococcus aureus* (*S. aureus*) and non-*aureus* staphylococci (NAS) and non-*aureus* staphylococci and mammaliicocci (NASM) [30]. *S. aureus* (a coagulase-positive spp.) is primarily but not exclusively responsible for CM, whereas NAS and NASM account for more than 50 % of SCM cases. Common NAS species include *S. epidermidis*, *S. caprae*, *S. simulans*, *S. chromogenes*, and *S. xylosus*, and NASM includes *M. sciuri*, *M. lentus*, and *M. vitulinus* [30].

**Fig. 1:**
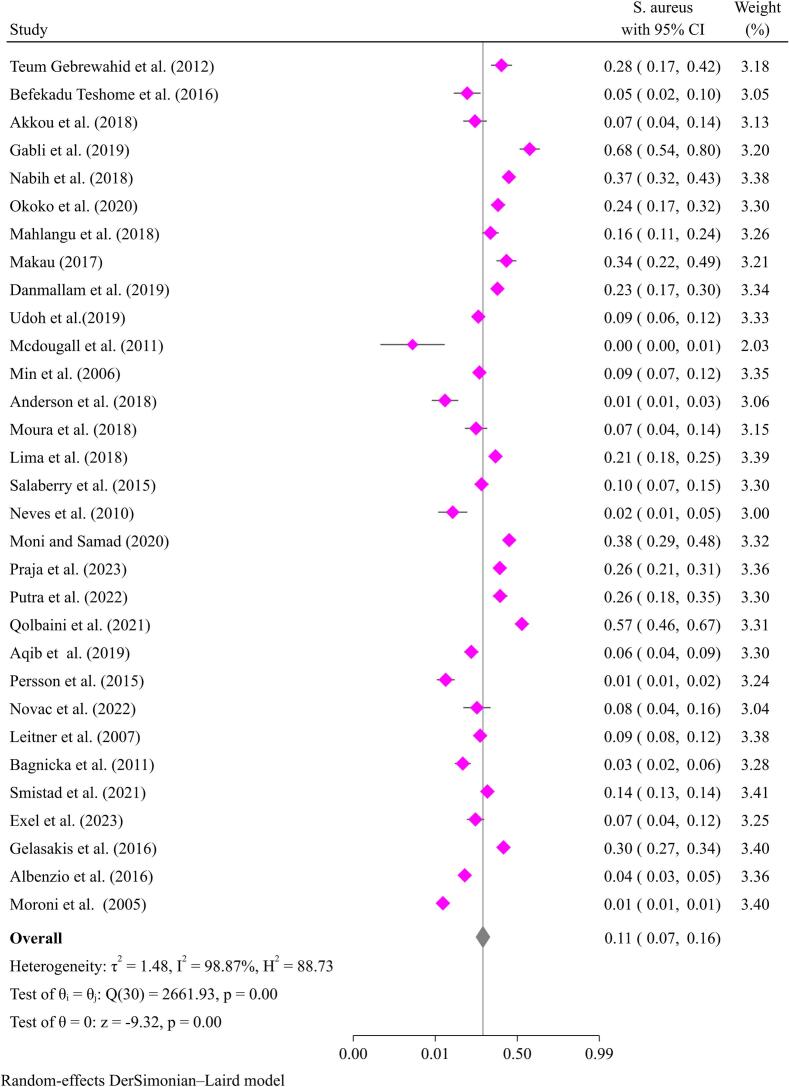
Pooled prevalence of mastitis in goats due to *Staphylococcus aurues.*

**Fig. 2:**
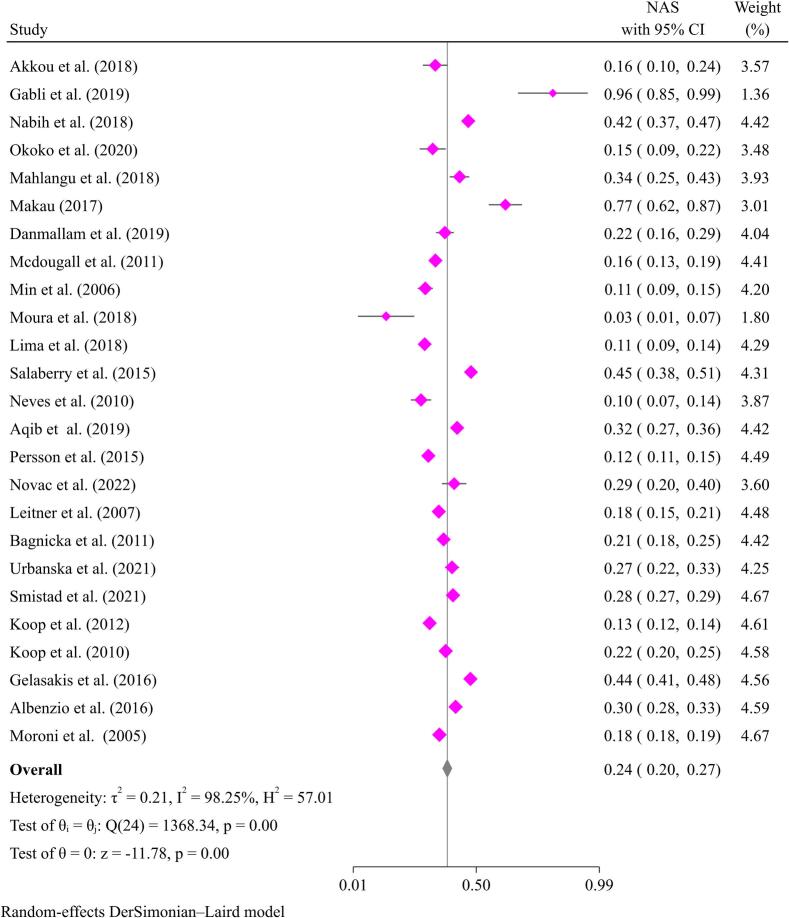
Pooled prevalence of mastitis in goats due to NAS.

A pooled prevalence of 11.0 % (95 % CI: 7–16 %) for *S. aureus* and 24.0 % (95 % CI: 20–27 %) for NAS was estimated from studies reported in goats (Fig. 1, Fig. 2). The prevalence of mastitis due to staphylococci in different studies varies from 1.0 % [[31], [32]] to 68.0 % [33].

**Fig. 3:**
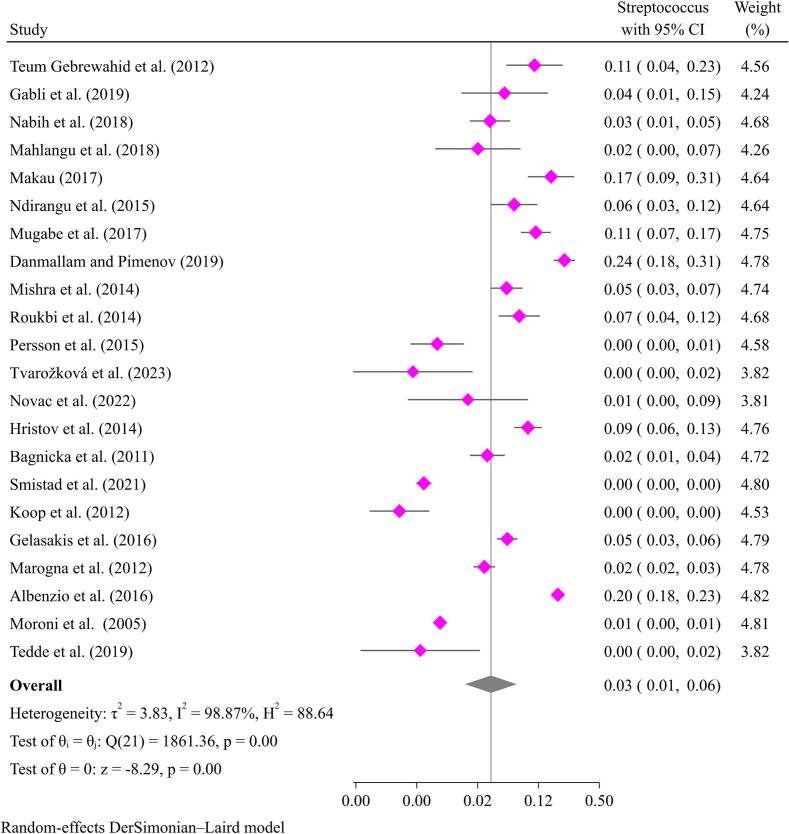
Pooled prevalence of mastitis in goats due to *Streptococcus* species.

*Streptococcus* is the other most common gram-positive isolate from mastitis in goats. Common species include *Streptococcus agalactiae* (*S. agalactiae*), *Streptococcus dysgalactiae* (*S. dysgalactiae*), *Streptococcus uberis*, and *Streptococcus pneumoniae*. *S. agalactiae* is responsible for CM, while *S. dysgalactiae* and *S. uberis* are associated with SCM in goats [34]. *S. agalactiae causes* CM, while *S. dysgalactiae*, *Streptococcus uberis*, and *Streptococcus pneumoniae* cause SCM in goats. The pooled prevalence of streptococcal mastitis in goats was synthesized to be 3.0 % (95 % CI: 1–6 %) (Fig. 3). Some studies reported mastitis caused by streptococci prevalence less than 1 % [22], while others reported as high as 24.0 % [16] (Fig. 3).

**Supplementary Fig. S1:**
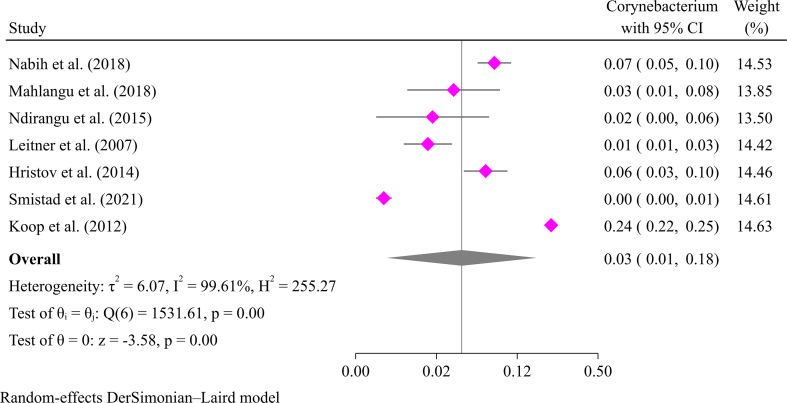
Pooled prevalence of mastitis in goats due to *Corynebacterium.*

*Corynebacterium bovis* and *Corynebacterium pseudotuberculosis* were identified in mastitic goats; the former is more common in cows than in goats. *Corynebacterium pseudotuberculosis* causes caseous lymphadenitis, a common condition in goats. The estimated pooled prevalence of mastitis in goats caused by *Corynebacterium* was 3 % (95 % CI: 1 % to 18 %) (Supplementary file Fig. 1). *Corynebacterium pseudotuberculosis* had a prevalence of 7.0 % in Egypt, according to Nabih et al. [33].

Mastitis caused by *Mycoplasma* spp., such as *M. mycoides* and *M. putrefaciens*, occasionally results in severe mastitis outbreaks in Europe. *Actinomyces*, *Actinobacillus*, *Micrococcus*, and *Mycobacterium* are also known to cause mastitis in goats. *Enterococcus* species, including *Enterococcus faecalis* and *Enterococcus faecium*, are among common causes of environmental mastitis.

**Fig. 4:**
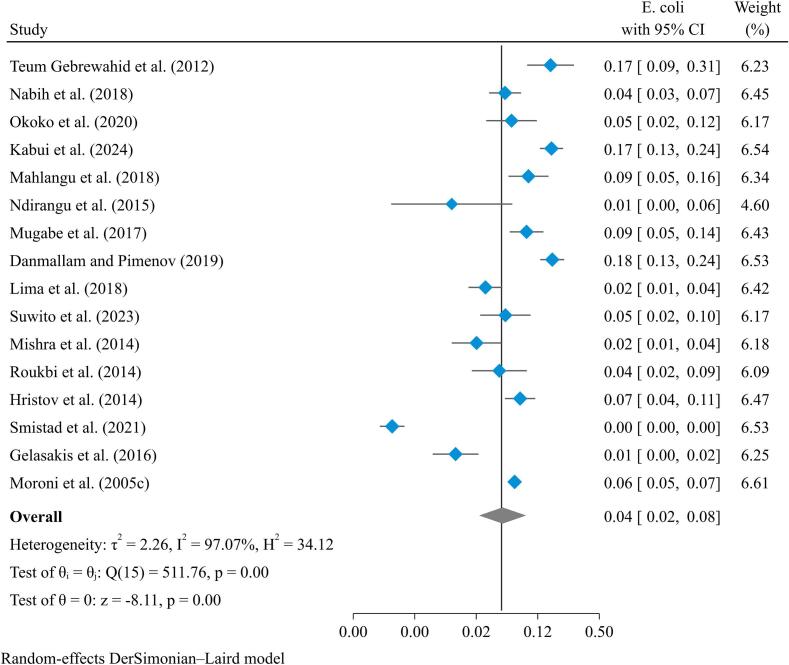
Pooled prevalence of mastitis in goats due to *E. coli.*

**Supplementary Fig. S2:**
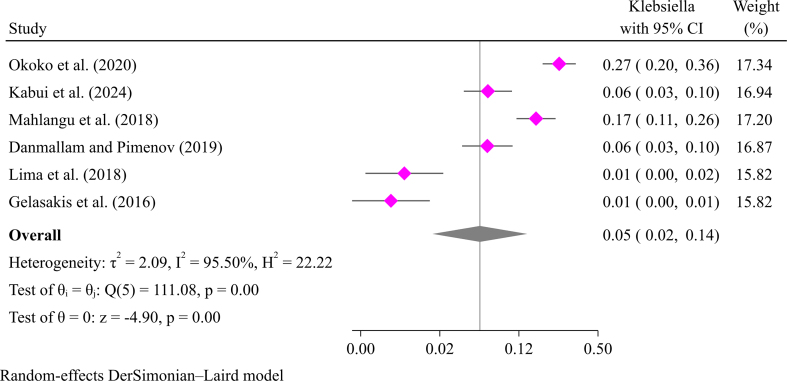
pooled prevalence of mastitis in goats due to *Klebsiella.*

Mastitis in goats is also caused by gram-negative bacteria like *E. coli* and *Klebsiella* species. *E. coli* is the most common cause of mastitis in goats among gram-negative bacteria [35]. A meta-synthesis indicated that *E. coli* causes goat mastitis with a prevalence of 4.0 % (95 % CI: 3.0–7.0 %) (Fig. 4). The pooled prevalence of other gram-negative bacteria such as *Klebsiella* (5 %; 95 % CI: 2–14 %), *Pseudomonas* (3 %; 95 % CI: 1–9 %), and *Citrobacter* (3 %; 95 % CI: 1–2 %) is shown in Supplementary file Fig. 2, Fig. 3, Fig. 4. Other gram-negative bacteria include *Pseudomonas*, *Citrobacter*, *Pantoea*, *Salmonella*, *Trueperella*, and *Serratia* [[16], [36]].

### 4.4 Viral causes

Although viral mastitis is less common than bacterial mastitis, goats are susceptible to certain viruses that can cause udder infections. *Caprine arthritis-encephalitis virus* (CAEV) causes indurative mastitis in goats [37], while the *Maedi-Visna virus* can cause rare SCM [38]. Foot and mouth disease, bluetongue, and peste des petits ruminants can also cause viral mastitis [39,40]. *Herpes virus*-induced papilloma is a rare benign mammary gland neoplasm. Furthermore, *poxvirus* and *enterovirus* can cause mastitis in goats [41].

### 4.5 Fungal causes

Mastitis can also be caused by fungal species such as *Aspergillus*, *Fusarium chlamydosporum*, *Histoplasma*, and *Cryptococcus*, which have a public health concern, particularly in regions where raw milk or unpasteurized milk consumption is common [[42], [43]]. *Candida* species (*Candida kefyr*, *Candida guilliermondii*, *Candida famata*, *Candida glabrata*, and *Candida albicans)* are isolated from mastitis in goats [44]. *Nocardia farcinica*, *Candida albicans*, *Cryptococcus neoformans*, and *Aspergillus fumigatus* were isolated from mastitis in goats [45].

### 4.6 Parasitic causes

Mastitis caused by parasites in goats is relatively uncommon in general. However, there were reports of *Toxoplasma gondii* associated mastitis. Tick infestations in poorly managed goats cause traumatic mastitis [46].

## 5 Risk factors associated with mastitis

Mastitis is a complex disease associated with a number of factors. Mastitis risk factors include host, environmental, and pathogen, with the pathogen factors being discussed in the etiology section.

### 5.1 Host factors

Host factors such as parity, genetics, kidding type, age, milk yield, and lactation stages impact udder health indicators in goats. These factors create uncertainty in sensitivity and specificity for udder health indicators and make it challenging to establish universal threshold values for different tests, which complicates mastitis monitoring in goats [11].

#### 5.1.1 Breed

According to breed-based comparative studies conducted in Kenya and Nigeria, the prevalence of mastitis in goats varies by breed [34,47]. A study in Ethiopia also found that the Begait breed had a higher prevalence of mastitis than the Abergelle breed. Differences in mastitis prevalence among breeds may be because some breeds produce more milk, leading to increased pressure in mammary glands and cisterns for milk storage. Goats that produce higher milk yields are at a higher risk (e.g., Begait) of developing mastitis. Besides, exotic or crossbred goats are more susceptible than indigenous or local breeds. This is because exotic or crossbred breeds may encounter unfamiliar environmental conditions and infectious agents, while indigenous breeds have built up a resistance to endemic pathogens.

#### 5.1.2 Parity

Various research findings revealed that parity is strongly associated with mastitis in goats. Primiparous goats produce and store less milk than multiparous goats, which reduces their vulnerability to mastitis. According to Gelasakis et al. [36], Mahlangu et al. [34], and Abdul et al. [48], goats in their latter parities have a higher incidence of mastitis. Goats in their third parity are at a higher risk of mastitis compared to those in their second parity, with first-time parity goats being the least affected [18]. Several researchers have also reported a positive relationship between parity and SCC [22]. This situation is associated with goats in later parities experience increased stress in their mammary glands due to multiple pregnancies and kidding.

#### 5.1.3 Age

Older goats are more vulnerable to mastitis than younger goats due to weakened immune function, increased tissue damage, and changes in mammary gland shape. Over time, mammary tissue grows and remodels, milk yield and storage capacity rise, and the teat end dilates, thus increasing the risk of bacterial infection. Mastitis prevalence could also be linked to the recurrence or persistence of previous infections [[36], [49]].

#### 5.1.4 Lactation stages

The SCC in goat milk increases as lactation progresses [50]. The mammary gland experiences an increase in leukocyte cells as the dry-off phase approaches. Specifically, neutrophils increased from 52 % in the first lactation to 69 % in the last lactation. After 90 milking days, milk somatic cells increase on average by 3.02 units per day to dry off. Thus, SCC in milk correlates inversely with lactation stage, making it difficult to distinguish between infected and healthy goats near the end of lactation [51]. At the end of lactation, mammary involution occurs, causing the udder to shrink, and keratin forms in the teat orifice to prevent pathogen entry. From this point onward, the incidence of mastitis is low until the next lactation. During the next early lactation, the incidence increases, especially contagious mastitis in machine milking, whereas environmental mastitis occurs whenever bedding hygiene is inadequate [15,34,52].

#### 5.1.5 Other host factors

The number of offspring influences the occurrence and severity of mastitis. A goat with multiple kids exhibits more compromised udder health than a goat with a single kid. The mastitic goat's uninfected udder half had lowers SCCs than the infected half but higher than non-infected goats' udder halves. Besides, the shape of teats and ends, as well as the length and circumference of the udder, affect mastitis vulnerability [53]. Goats with longer teats are more susceptible to mastitis than goats with shorter teats; a positive correlation has been found between udder length and udder health. However, there are contradictory reports, such as Khasanah et al. [54], who found a negative correlation between udder circumference and udder health.

### 5.2 Environmental and management risk factors

Physical trauma (cuts, bruising, deep punctures), dryness, excessive udder massaging, and failure to milk on time are all risk factors for mastitis. Nutrition is one of the risk factors for mastitis; goats with low energy levels are more vulnerable. Hormonal imbalances and environmental irritants can also contribute to its occurrence. Inadequate cleaning or an unhygienic udder often contributes to decay, milk stasis, and non-natural milk secretion and ejection [16].

Mastitis, especially when caused by minor pathogens, requires some external environmental risk factors. Temperature fluctuations are one of the environmental risk factors for mastitis, as extreme heat or cold alters blood flow to mammary tissues, impairing immune function. Abrasive bedding, insects (flies), or chemical irritations disrupt teat skin integrity, enabling less pathogenic microorganisms to colonize the udder via environmental splashing or contact with an infected udder. Mismanagement risk factors in dairy farming include improper milking techniques, cross-contamination, and handling multiple animals without post-milking sanitation [23]. Environmental mastitis requires such poor management practices as improper milking, unhygienic bedding, and contaminated feed/water troughs. The lack of specific regulations and diagnostic thresholds for mastitis tests in goats has led to increased public health risks but decreased farmer motivation for routine testing [55,56].

## 6 Diagnosis of mastitis

### 6.1 Diagnosis of CM

Clinical mastitis is a rare but serious problem for dairy farmers. It can be acute or chronic, and bacteria tend to be the primary cause. To diagnose CM, it involves observation of inflammatory signs and other general signs of illness, such as decreased feed intake and lethargy. A physical examination of the mammary gland may reveal signs of inflammation, such as heat, firmness, and pain. In addition, changes in milk texture and appearance are important indicators. The milk is lumpy and smells unpleasant. The goat is reluctant to milk its kid and refuses to let it suck. These clinical presentations suggest a serious case of udder health issues (CM) that necessitates immediate attention [[11], [57]].

### 6.2 Diagnosis of SCM

Subclinical mastitis is the most prevalent type of mastitis, increasing the risk of CM and zoonotic diseases when unprocessed or inadequately processed milk and dairy products are consumed. Effective management and control of SCM depend on regular diagnostic testing and the implementation of appropriate management strategies. Various diagnostic techniques, including SCC, CMT, white side test (WST), pH measurement, milk electrical conductivity test (EC), and bacterial isolation and identification, are widely utilized [48,34]. The reliability of some of these diagnostic tests for SCM in goats is questionable, as SCCs in non-mastitic goats frequently exceed the established normal range for cows.

Somatic cell count (SCC).

Somatic cells, mainly white blood cells, are part of the body's defense system present in normal milk; however, a rise in their levels may suggest an inflammation of the mammary gland [58]. However, the reliability of SCC for SCM diagnosis in goats is questionable, as somatic cells in healthy goats exceed the recognized normal range for cows [59]. Tedde et al. [60] found that SCC in goats detects IMI at a sensitivity of 71.43 % for SCC > 0.5 × 10^6^ cells/ml and 23.23 % for SCC > 1 × 10^6^ cells/ml. In goat milk, neutrophil levels range from 45 % to 75 %, while in cow milk, the range is 2 % to 20 %. Consequently, there is no universally accepted single cutoff point for SCC to detect mastitis in goats, resulting in disparities in regulatory standards and scholarly references. The US has set a maximum SCC cutoff of 1.5 × 10^6^ cells/ml for goats, nearly four times the limit for cattle. However, many countries do not have such established standard regulatory limits. Varied SCC limits had been used by scholars, such as 500,000 cells/ml [61], 1 × 10^6^ cells/ml [22], and 2 × 10^6^ cells/ml [[62], [63]].

#### 6.2.1 California mastitis test (CMT)

The California mastitis test is the cheapest and most widely used diagnostic test for SCM in dairy animals, including goats [15]. This simple and cost-effective test is critical for ensuring milk quality and safety [64]. The CMT reagent works by disrupting somatic cell membranes, which subsequently bind to cellular DNA. When there are enough somatic cells, the interaction causes noticeable changes in milk consistency, increasing viscosities. Thus, the SCC determines the thickness of gel formation [64]. The reaction's intensity is subjectively judged, ranging from 0 to +3, as well as 0 to +4 [34].

Although CMT is the most widely used test, inexpensive, simple to perform, and requires no prior experience or advanced training from farmers, its low specificity for diagnosing IMI in goats remains a major limitation. As discussed in the SCC section, milk SCCs in goats are affected by a variety of factors other than inflammation. The CMT test can only detect 80 % of IMI cases if milk somatic cells exceed 7 × 10^6^ cells/ml or 62 % if somatic cells exceed 5 × 10^6^ cells/ml. This reduces the CMT test's reliability and raises questions about its validity. This reduces the CMT test's reliability and raises questions about its validity. CMT as an initial screening test can be useful; however, confirmatory tests are strongly advised for better diagnosis, and CMT alone should not be used to make decisions that could jeopardize a dairy farm's survival and profitability.

#### 6.2.2 Modified white side test (WST)

The Whiteside test (WST) is a somatic cell precipitating diagnostic procedure using sodium hydroxide. The precipitate is graded on a scale of 0 to +4, with greater precipitation corresponding to higher milk somatic cell concentrations [64]. This diagnostic test is not as widely used as SCC and CMT, so it has limited application in SCM diagnosis.

#### 6.2.3 Milk electrical conductivity (EC) test

Electrical conductivity is a parameter that measures a substance's ability to conduct electricity. Ions from sodium, potassium, chloride, and calcium have an effect on milk's EC. Mastitis caused by infection (IMI) raises ion levels in milk (due to increased tissue permeability), resulting in increased EC. Several scholars [17] evaluated this method's applicability in SCM diagnosis in goats and reported that its sensitivity and specificity are dependent on factors such as severity, infection stage, prevalence, and cause.

#### 6.2.4 Bacteriological identification

Bacteriological isolation and identification is regarded as the gold standard for mastitis diagnosis. However, bacterial identification procedures are labor-intensive and need specialized facilities, making regular implementation difficult in low-income countries with under-equipped laboratories. Thus, compared to bacteriological testing on each sample, targeted bacterial identification from a prescreened sample can significantly reduce costs, labor, and errors. Recent research suggests that rapid farm tests, such as MicroMast™ and ClearMilk Test, can accurately diagnose mastitis at the farm level with high sensitivity and specificity [8]. On-farm culture is a system that allows veterinarians to advise producers on the best strategic treatment options for clinical mastitis cases without the delay that laboratory culture causes between milk sample submission and results reporting [65].

Minor bacterial pathogens can cause infection but not significant concern, and the bacterial colony unit evaluations have paramount importance. Some minor bacteria can cause inflammation without causing significant damage or posing a public health risk, so the bacterial colony forming unit count (cfu) is an important assessment. The EU has a maximum limit (1.5 × 10^6^ cfu/ml) for goat milk, and farms that do not meet it face price reductions [66,67].

#### 6.2.5 Molecular techniques

A key component of the polymerase chain reaction (PCR) assay process is the successful isolation of DNA from samples, which can then be detected via nucleotide-specific primers. Primers have been designed to identify specific genes at various loci of presumed pathogens and differentiate their pathotypes and serotypes. In theory, with appropriate DNA extraction techniques and a sufficient level of DNA purity, PCR methods can detect a single DNA molecule, which can be amplified to yield a greater quantity of DNA for further analysis. According to Smistad et al. [22], the qPCR *S. aureus* identification had very high sensitivity and specificity SCC.

## 7 Treatment, control and prevention of mastitis

Dry-period therapy can reduce milk discarding, control latent infections, and reduce the risk of antimicrobial resistance and residues in milk, making it a viable treatment option. Selective dry period therapy based on the risk of having an IMI during dry-off, which is a major risk factor for mastitis during kidding and early lactation, is a cost-effective, prudent AM use strategy. Furthermore, routine mastitis diagnosis and early treatment can significantly reduce future financial losses while improving welfare [68].

There have been no successful controlled trials to show that a single route therapy (parenteral vs. intramammary) is effective for the treatment of all types of mastitis [69]. Parenteral antimicrobial administration has higher drug bioavailability, making it more advantageous and appropriate for both systemic and mild local intramammary infections. Intramammary infiltration, on the other hand, results in high local drug concentrations, which may be appropriate when local circulation is disrupted. More importantly, identifying the pathogen and conducting antimicrobial resistance testing are critical for prudent, cost-effective antimicrobial selection decisions and improved mastitis control. Increased veterinary drug use and culling will not always increase farm return or profit unless cost-effective judicious AM are used. In general, infections due to minor pathogens had higher cure rates compared to those by major pathogens [70].

The prevalence of infectious mastitis can be reduced through prudent prevention measures, improved management, and sanitation [56]. Keeping udders clean and practicing hygienic milking are critical for the prevention of mastitis. Teat dipping (before and after milking) and hand washing reduce mastitis cross-transmission. Controlling respiratory diseases in kids (via vaccination and temporary isolation), culling animals with recurrent infections, and maintaining clean bedding and milking equipment can contribute to further transmission control [23,21]. It is also critical to limit activities like excessive udder massaging, machine stripping, and teat cup reattachment. Starting milking with a first-parity and healthy goat has advantages to prevent further infection spread [71].

## 8 Economic impact of mastitis in goats

Mastitis has a significant negative impact on dairy production, compromising milk yield and quality, market supply, and pricing. Contagious agalactia (CA) is a serious disease that threatens dairy goat farms; all animals might be affected, and milk yield can drop by 25 % to 100 %. Severe mastitis cases may cause death to the goat and/or spread the infection to the kids [53]. Mastitic goats could have compromised health and production performance that would require intensive labor and veterinary care. Blanket dry period therapy, used in intensive production systems to treat all animals and udder halves regardless of infection status, is viewed as a tax on dairy farms. In addition, discarding milk due to poor quality and drug withdrawal time are both undesirable [53]. A lack of awareness and failure to detect mastitis early on can lead to chronic infection, clogged teats, and jeopardized future lactation.

SCM is more economically significant than CM because it occurs 15–40 times more often. CM has a higher treatment cost per animal but a lower cure rate than SCM. Another important negative consequence is the early culling of productive animals [53]. Despite its low prevalence and sporadic nature, CM has severe economic and public health consequences, including a significant decrease in milk yield or complete cessation. In intensive production systems, CM is the leading cause of early culling. The economic costs of unseen milk reduction, discard due to poor milk quality, and public health risks are enormous consequences of SCM. Koop et al. [49] reported that SCM caused by major pathogens results in 0.13 kg/day and 0.29 kg/day milk loss at the early and late lactation stages, respectively.

Mastitis in dairy cows causes an estimated $35 billion in economic losses globally each year [64]. Dairy cow losses total $2 billion in the United States and $526 million in India each year [72]. In Ethiopia, this can cost as much as $2949.80 (Belay [73]). However, estimating the economic loss in goats is challenging due to insufficient research attention, limited goat milk marketing practices, and the frequent oversight of mastitis in goats.

## 9 Public health concerns associated with mastitis

Humans and animals coexist with microorganisms, but only a small number of them cause diseases. About 70 % of emerging diseases and 60 % of human infectious diseases are zoonotic. Additionally, 80 % of infectious diseases can infect multiple hosts, which contributes to the emergence of new diseases. Bacteria are responsible for 52 % of zoonotic diseases [74,75].

Intramammary infection has significant implications for food safety and public health. Bacteria present in milk due to IMI can alter its physicochemical properties, including protein, fat, lactose, and immunoglobulin levels. This alteration affects the production of various milk products, such as cheese, whey, and yogurt, as well as the nutritional content necessary for human consumption. Microorganisms and toxins from IMI may cause potentially fatal infections in humans. If milk or milk products are positive for zoonotic bacteria such as *Staphylococcus*, *Streptococcus*, *Mycobacterium*, *Salmonella*, *Listeria*, *Campylobacter*, *E. coli*, and *Klebsiella*, public health can be endangered. Biological toxins present in IMI milk include aflatoxin (fungal), pyrogenic toxins, enterotoxins, and Shiga toxins [76]. Antimicrobial residues from mastitic goats' milk can be transferred to humans in small doses [34], potentially causing allergic reactions, gastrointestinal intolerance, and cancer in consumers. Furthermore, a low dose in humans raises the risk of antimicrobial resistance [77,78].

Zoonotic diseases have significant impacts on goat farmers whose livelihoods depend on milk and products; they are less literate, have limited knowledge, attitudes, practices regarding mastitis, and consume mostly unprocessed or inadequately processed milk. Mastitis prevention and control in goats require the involvement of both veterinarians and farmers; thus, both veterinarians and farmers serve as stewards of animal welfare and production, as well as milk quality and public health. One of the most serious issues in the healthcare system is specialization, which isolates people from the network and discourages the exchange of common ideas and practices. To address recent animal, human, and environmental issues, a collaborative network of diverse disciplines is required. The One Health approach has gained popularity in recent zoonotic outbreaks such as COVID-19, Ebola, and Middle East respiratory syndrome.

## 10 Antimicrobial resistance

The discovery of antimicrobial agents was a monumental breakthrough in medical history. Antimicrobials (AM) are frequently used in livestock production for therapeutic, preventive, and growth-promoting purposes [79]. AM are magical bullets that can cure a wide range of bacteria, parasites, and fungal infections.

However, excessive dependence on AM has fueled antimicrobial resistance (AMR), one of the world's growing health threats, estimated to cause nearly 5 million human deaths each year [80], with the figure expected to rise to 10 million by 2050 [81]. AMR is a condition in which medications no longer work to treat infections caused by bacteria, viruses, fungi, or parasites. Misuse of AM, combined with a lack of new drug discovery, is contributing to the natural imbalance among microorganisms, plants, animals, environments, and humans [82]. Microorganisms are developing AMR mechanisms, rendering conventional antimicrobials ineffective for treating human and animal diseases [83]. This reality, combined with unregulated widespread misuse of antimicrobial agents for treating infections, puts at risk healthcare systems and life in general.

Microorganisms can evade attacks from AM and humoral cells by developing protective mechanisms. AMR development mechanisms include structural and genetic changes, reduced cell membrane permeability, and the production of biofilms, enzymes, or toxins [77]. Several bacteria acquired AM-resistant genes. Bacterial biofilms are three-dimensional protective polymeric matrix structures (metabolic products of sugar and protein) that surround bacterial cells and render them resistant to antibiotics and the immune system. Some of the major pathogenic bacteria with this characteristic include *Staphylococcus*, *Enterococcus*, *Listeria*, *Streptococcus*, and *Pseudomonas* [25,26].

Several studies have been conducted on the AMR trends of different bacterial species. *Staphylococcus* spp. was found with resistance to a variety of AMs, including amoxicillin, oxacillin, vancomycin, tetracycline, and penicillin [84,34]. *Streptococcus* spp. have also developed resistance to ampicillin, amoxicillin, and tetracycline [68], while *E. coli* was resistant to penicillin, tetracycline, and chloramphenicol [34].

Food animals are the primary source of AMR microorganisms for both humans and the environment. More than half of drug doses administered to animals are excreted unchanged in urine and feces, which can impact soil and crop microbial communities [85]. The increasing incidence of animal-origin emerging diseases and AMR underscores the need for collaboration and networking among different disciplines for urgent decisions and solutions. It is critical to address zoonosis at the intersection of human, animal, and environmental health concerns. Managing mastitis with prudent, judicious AM uses to improve production not only safeguards public health but also underscores the crucial role of veterinarians as primary stewards of human health, expanding their influence beyond livestock health and production.

## 11 Prevalence of mastitis in goats

Before 20 years ago, the pooled estimated prevalence of mastitis in goats was reported to be between 30 % and 50 %; however, this data needs to be updated for the current context. Although there are recent comprehensive meta-analyses for mastitis in cows and buffaloes, similar studies for goats have not been conducted yet. Only some national attempts have quantified mastitis prevalence in goats with other species. In Brazil, Acosta et al. [86] systematically reviewed and found that the prevalence of SCM was 48.6 % in cows, 30.7 % in goats, 31.45 % in sheep, and 42.2 % in buffalo. A similar review by Nuraini et al. [87] in Indonesia found mastitis prevalence was 63.4 % in cows and 44.9 % in goats. A review by Hasan [88] in Bangladesh also found a prevalence of mastitis in cows at 43.0 % and goats at 31.0 %.

**Fig. 5:**
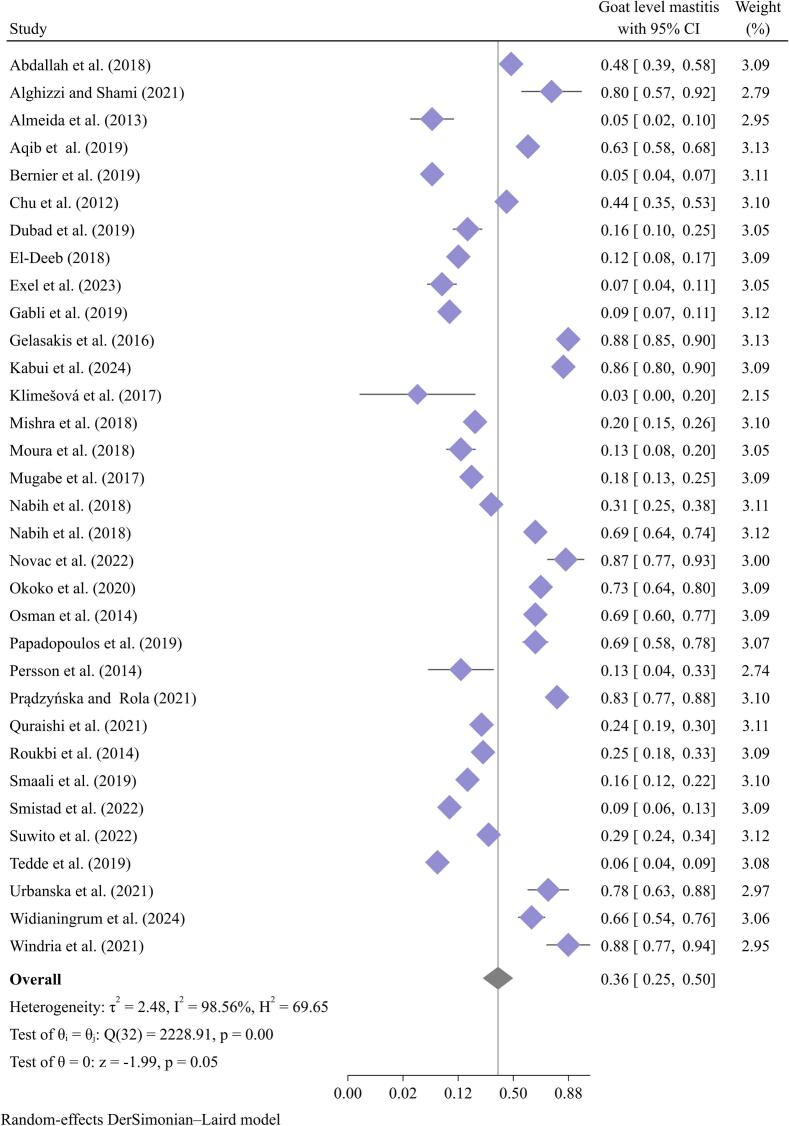
Pooled prevalence of mastitis at goat level.

**Fig. 6:**
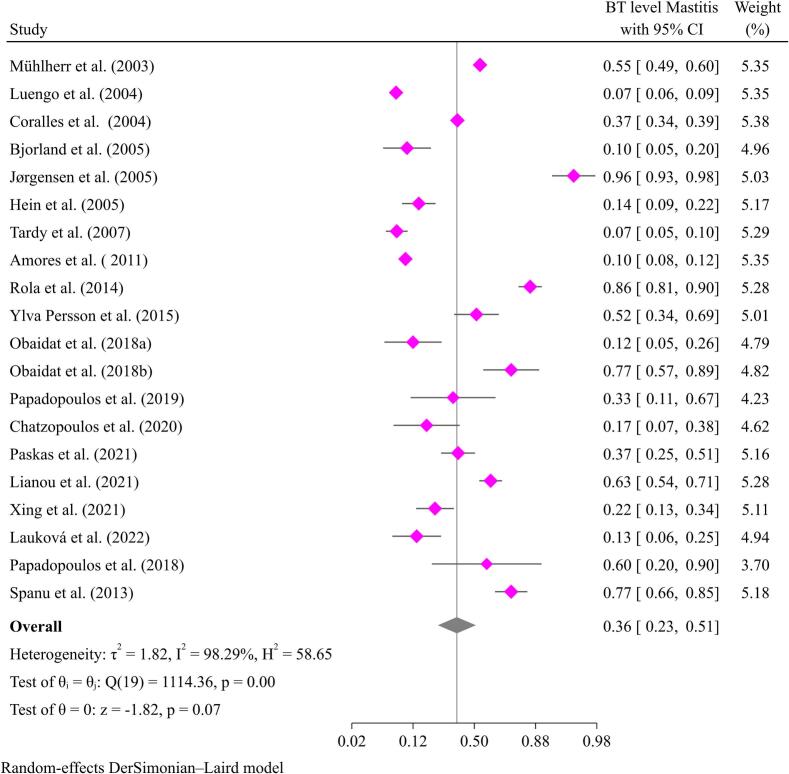
Pooled prevalence of mastitis at bulk tank milk (BT).

The meta-analysis in this review found mastitis prevalence of 36 % (95 % CI: 25–50 %) at the goat level and 36 % (95 % CI: 23–51 %) in bulk tank milk, as shown in Fig. 5, Fig. 6. Many independent studies have reported goat mastitis prevalence below 10 % in various countries, including the Czech Republic [89], Italy [60], the Netherlands [90], and the USA [66]. This review shows that the prevalence of mastitis remains consistent at a range of 25–50 %, similar to estimates from 20 years ago. As a result, it seems that the interventions implemented have not been effective in controlling or preventing this condition.

Studies in Norway [22], and Algeria [91], have reported mastitis prevalence ranging from 10 % to 20 %. In contrast, higher mastitis prevalence rates exceeding 70 % have been documented in diverse regions such as Kenya [[92], [93]]; [93]), Greece [94], Poland [95,37], and Saudi Arabia [96], which consistently report these elevated figures. This suggests that region-specific risk factors, such as climatic stress, intensive farming practices, or gaps in veterinary care, may influence the prevalence of mastitis.

## 12 Conclusion

This review investigated how mastitis was a major constraint on goat milk production around the world. The pooled prevalence of studies found a higher incidence of mastitis and identified major pathogens. Due to limitations in test accuracy, it may be difficult to distinguish between sick and healthy goats, potentially leading to a misdiagnosis of SCM. CM and SCM result in significant economic losses, with SCM having the most impact. The most commonly reported bacteria associated with mastitis are *Staphylococcus*, *Streptococcus*, and *E. coli*. These bacteria are recognized as important zoonotic pathogens causing a variety of human illnesses. The high prevalence of *Staphylococcus* proved poor mastitis management methods. Despite the fact that goats are critical milk sources for impoverished communities in tropical regions, mastitis research remains disproportionately focused on cattle, a gap that must be filled urgently through targeted studies. Furthermore, the dairy industry requires collaborative One Health initiatives to address public health concerns at the animal-environment-human interface.

The following are the supplementary data related to this article.

**Supplementary Fig. S3:**
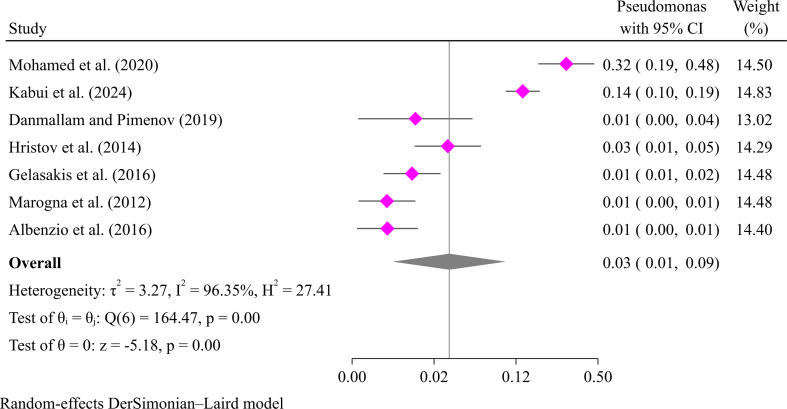
Pooled prevalence of mastitis in goats due to *pseudomonas.*

**Supplementary Fig. S4:**
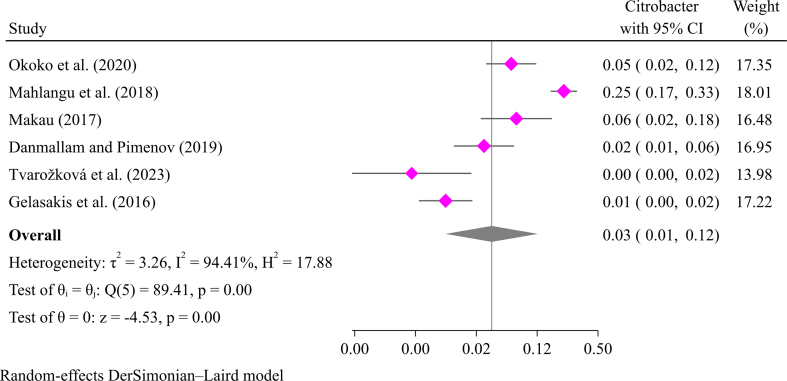
Pooled prevalence of mastitis in goats due to *Citrobacter.*

## Funding

This research received no specific grant from any funding agency in the public, commercial, or not-for-profit sectors.

## Availability of data and materials

Data will be shared up on responsible request to the corresponding author.

## Declaration of competing interest

This review examines existing studies and does not involve new data collection or direct interaction with participants. As such, it is exempt from ethical approval and consent requirements, in line with standard research guidelines.

All authors declare that they have no competing interests related to this study.

## Data Availability

Data will be made available on request.
